# Water molecular structure underpins extreme desiccation tolerance of the resurrection plant *Haberlea rhodopensis*

**DOI:** 10.1038/s41598-019-39443-4

**Published:** 2019-02-28

**Authors:** Shinichiro Kuroki, Roumiana Tsenkova, Daniela Moyankova, Jelena Muncan, Hiroyuki Morita, Stefka Atanassova, Dimitar Djilianov

**Affiliations:** 1 Laboratory for Information Engineering of Bioproduction, Graduate School of Agricultural Science, Kobe University, 1-1 Rokkodai, Nada, Kobe 657-8501 Japan; 2 Biomeasurement Technology Laboratory, Graduate School of Agricultural Science, Kobe University, 1-1 Rokkodai, Nada, Kobe 657-8501 Japan; 3 Abiotic stress, AgroBioInstitute, Agricultural Academy, 8 Dragan Tzankov Blvd., 1164 Sofia, Bulgaria; 4 Nanolab, Biomedical Engineering, Faculty of Mechanical Engineering, University of Belgrade, Kraljice Marije 16, Belgrade, 11120 Serbia; 5 NIRECO CORPORATION, 2951-4, Ishikawa machi, Hachioji, Tokyo Japan; 6 Department of Biochemistry, Microbiology and Physics, Faculty of Agriculture, Trakia University, Stara Zagora, Bulgaria

**Keywords:** Drought, Near-infrared spectroscopy

## Abstract

*Haberlea rhodopensis* is a resurrection plant with an extremely high desiccation tolerance. Even after long periods of almost full desiccation, its physiological functions are recovered shortly upon re-watering. In order to identify physiological strategies which contribute to its remarkable drought stress tolerance we used near infrared spectroscopy to investigate the state of water in the leaves of this plant and compared it to its relative, non-resurrection plant species *Deinostigma eberhardtii*. Here we show, using a novel aquaphotomics spectral analysis, that *H*. *rhodopensis* performs a dynamic regulation of water molecular structure during dehydration directed at drastic decrease of free water molecules, increase of water molecules with 4 hydrogen bonds, and a massive accumulation of water dimers in the full desiccation stage. Our findings suggest that changes in water structure mirror the changes in major metabolites and antioxidants which together constitute a robust defense system underlying the desiccation tolerance of the resurrection plant, while the water dimer may hold special importance for the “drying without dying” ability.

## Introduction

Water deficit is the most critical stress for the living organisms on our planet. Being sessile organisms without the option to physically leave the location of stress, plants developed various mechanisms to avoid, escape or to cope with dehydration^1^. The vegetative tissues of the majority of plants are highly sensitive to water deficit, losing viability upon loss of 41% to 70% (depending on the species) of total water content^2^. However, there is a small group of plant species, whose vegetative organs can survive extreme dehydration. They are known as resurrection plants because after long periods of almost full desiccation (~10% relative water content/RWC) they can recover fully and quickly upon rewatering^3^. This unique and still mysterious ability of the resurrection plants makes them useful model system for numerous studies on plant response to desiccation stress^4^.

Extreme water stress induces mechanical stress associated with turgor loss, oxidative stress from free radical-mediated processes and destabilization of macromolecular integrity^5^. Water loss of leaf tissues results in considerable anatomical and ultrastructural re-organization as well as various metabolic and physiological changes. Molecular and biochemical investigations indicated that desiccation tolerance is a complex phenomenon that appears to be a result of coordinated physiological and biochemical alterations at cellular and molecular level, including accumulation of various osmolytes, non-reducing sugars and late embryogenesis-abundant (LEA) proteins, as well as an existence of a strong antioxidant system – all directed at preventing oxidative damage and maintaining the structure of macromolecules and membranes^6–8^. At the core of many of these processes is water: a driving force for the assembly of phospholipids into biological membranes and conformation of proteins^6^. In fact, all cellular events could be related to the physical states of the cell-associated water^9–11^.

Measurement and characterization, *in vivo*, of water molecular structure and dynamics is however, a particularly challenging task^12^. Proton nuclear magnetic resonance (^1^H-NMR) spectroscopy and its imaging technique (MRI) have been widely used in recent years for studying water in living systems^12–15^, and especially for the examination of water status in fruits and vegetables in relation to their ontogenetic and physiological changes^12,13,15–19^. These techniques measure mobility of water molecules in the plant tissues, and how much this movement is restricted by surrounding biomolecules thus essentially providing an insight into hydrogen bonding and water structure in plant tissues.

Near infrared (NIR) spectroscopy is a proven method for the estimation of various quality parameters of plant tissues, such as mechanical properties (firmness, stiffness, and hardness) and chemical properties (soluble solid content, dry matter, acidity, pH, chlorophyll, etc.)^20^. The NIR spectra contain information about the light absorbance of molecular structures in the sample, at observed absorption bands which are overtone and/or combination bands of the fundamental vibrations of the chemical bonds appearing in the mid infrared region. The chemical bonds can be thought of as weak springs holding atoms together, which naturally vibrate with a certain frequency resulting in respective molecular spectra^21^. Different chemical bonds vary in strength and vibrate differently giving rise to differently looking spectra. Hence, any change in the molecular structure subsequently leads to spectral changes. In the NIR region, specifically, bond vibrations between oxygen and hydrogen (OH), carbon and hydrogen (CH) and nitrogen and hydrogen (NH) can be observed. Being non-destructive and requiring only minimal to no sample preparation, NIR spectroscopy can be a useful tool for studying molecular changes in the desiccation-tolerant plants.

Recently introduced, a novel scientific discipline of aquaphotomics expanded applications of near infrared spectroscopy to more in-depth exploration of water, its structure and roles in biological systems^10,22^. Although focused on measurement and characterization of water molecular network and its dynamics, aquaphotomics provides indirect characterization of the biological system as a whole, where biomolecular dynamics is mirrored by subtle changes in the water matrix^10,23^. This so-called *“water-mirror approach”* utilizes extremely high sensitivity of water’s hydrogen bonds which readily adapt to any change of the aqueous system and produce differences in its spectra which can be observed, measured, analyzed and interpreted. Application field of aquaphotomics is very wide and ranges from fundamental studies of water solutions to complex diagnostics in veterinary and human medicine^24^. Previously performed aquaphotomics studies on plants revealed substantially altered water structure in soybean leaves in very early stages after mosaic virus infection^25^ while studies on cold stress in soybean plants revealed that the changes in water structure were detectable even in the case of very mild cold stress conditions and further that different levels of cold tolerance in soybean cultivars were directly related to their ability to keep the water structure of the leaves in the non-hydrogen bonded state^26^. These works provided the rationale for further research into the processes underlying the plants’ stress response and the role of water in it.

To the best of our knowledge, the changes in the dynamic state of water molecular structure in plants during dehydration and subsequent rehydration process have not been investigated so far. Understanding the desiccation tolerance mechanism requires systems biology approach and combined efforts of genomics, transcriptomics, proteomics and metabolomics^27^, to which the complementary “omics” analysis of the most essential component of all living tissues – water, aquaphotomics, has to be introduced.

In our previous works we studied the dynamics of key components involved in leaf tissue antioxidant systems^28^ and performed metabolite profiling of more than 100 compounds^29^ during dehydration and rehydration of the resurrection plant *Haberlea rhodopensis* and the related, non-resurrection species *Deinostigma eberhardtii* (previously known as *Chirita eberhardtii*). The comparison strongly suggested that resurrection behaviour of *H*. *rhodopensis* is a result of a broad set of adaptations where accumulation of soluble sugars and strong antioxidants possibly play pivotal roles^28,29^.

In the present study near infrared spectroscopy and aquaphotomics multivariate statistical analysis were applied to investigate the dynamic state of water in the Balkan endemic resurrection plant species *H*. *rhodopensis* and the non-resurrection plant species *D*. *eberhardtii* from the same botanical family *Gesneriaceae* during desiccation and subsequent rehydration. The main objective was to discover water spectral changes related to the physiological status of the plants.

## Results

### Relative water content (RWC)

The overwhelming majority of higher plants are able to tolerate the loss of certain amount of water for relatively short period and to recover to their normal state upon rehydration. Usually they can survive relatively short periods at about 35–40% relative water content (RWC). After drying below this “point of no return” most plants are not able to resume their normal physiological activities despite the water availability.

**Figure 1:**
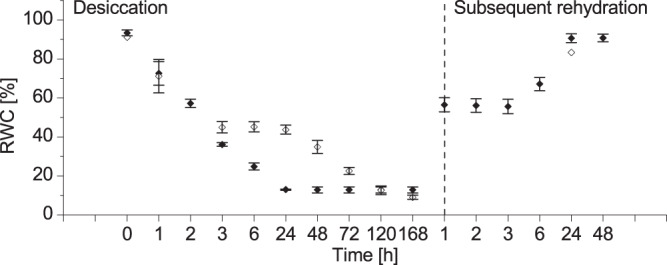
Relative water content (RWC %) dynamics during desiccation and subsequent rehydration. Changes in the relative water content (RWC %) during desiccation and subsequent rehydration in *Haberlea rhodopensis* (♦) and *Deinostigma eberhardtii* (◊) respectively. Error bars indicate the standard error of the mean for 3 experiments. When no error bar is visible, the standard error is less than the symbol. (For the link between exact sample timing and respective RWC content measured, see Supplementary Information, Table S1).

In this respect, under our experimental conditions *D*. *eberhardtii* plants make no exception (Fig. 1). As previously shown^28^ at removal from the culture vessels, plants have RWC close to that of the *in vitro* plantlets of *H*. *rhodopensis* at this stage (around 92%). When exposed to air drying, the water content of *D*. *eberhardtii* plants decreased gradually and relatively slowly reaching about 35% RWC in 48 h. This was the lowest RWC level at which *D*. *eberhardtii* plantlets still recover at rehydration. Similar to previous studies^28,29^, further air drying (up to 168 h) led to irreversible water losses. The rehydration of *D*. *eberhardtii* plantlets, performed on the specimens dried to about 35% RWC led to successful recovery, reaching ~83% RWC in 24 h.

The dynamics of RWC in *H*. *rhodopensis* under desiccation was different (Fig. 1). When removed from the culture vessels, the plantlets had ~94% RWC. Under air drying their water content decreased intensively, reaching ~13% RWC after only 24 hours. Further drying did not reduce the water content. Fully desiccated plantlets were maintained for 1 week under controlled conditions. Then, they were exposed to re-watering which immediately started their recovery. As in previous studies^28,29^ *H*. *rhodopensis* plantlets reached their control RWC values in 24 h.

### NIR Spectra– Initial Comparison

**Figure 2:**
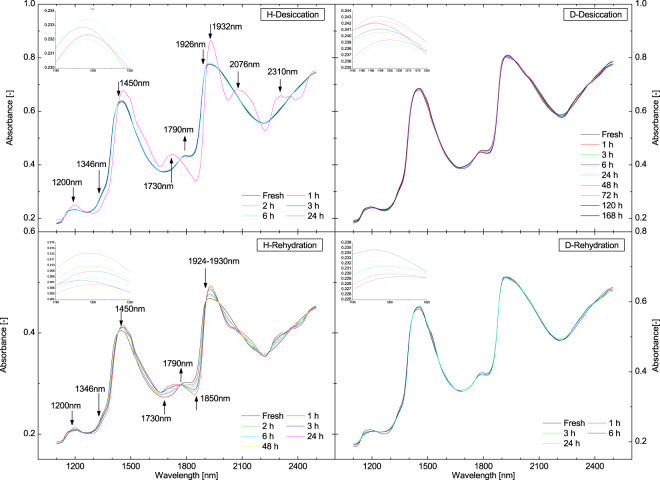
EMSC transformed spectra of plants’ leaves. EMSC treated spectra of *Haberlea rhodopensis* (left) and *Deinostigma eberhardtii* (right) during desiccation and subsequent rehydration.

EMSC treated and averaged NIR absorbance spectra of plants’ leaves during desiccation and rehydration stages are plotted in Fig. 2. Broad absorbance features were found for both plants and the shape of the spectra is typical and often found in spectra of plant food products with high water content^22,30–32^. Prominent absorbance features around 1200 nm, 1450 nm and 1930nm may be attributed to the combination of the first overtone of the O-H stretching and the OH-bending band, first overtone of the OH-stretching band and combination of the OH-stretching band and the O-H bending band of water, respectively^22,33–38^. These spectral features, arising from the water in the tissues, dominated the NIR spectra of both plants’ leaves.

Visual inspection of EMSC treated absorbance spectra of plants revealed major differences of plant species during desiccation and rehydration. In general, spectra of *H*. *rhodopensis* leaves showed much more variation during de- and re- hydration, contrary to *D*. *eberhardtii* whose spectra looked rather overlapped during the entire process. In *H*. *rhodopensis* spectra during desiccation to about ~25% RWC (6 h), an increase of absorbance was noticeable at 1450 nm and 1930nm, together with the shift towards longer wavelengths (i.e. red shift). The band at 1200 nm did not show clear tendency during desiccation (top-left inset, Fig. 2). In addition gentle decrease of absorbance was observed at 1346 nm and 1790nm. The spectral changes at 1346 nm and 1790nm are also related to the changes of water^39^ (Detailed assignments of all absorption bands observed in the NIR spectra of plants is provided in Supplementary Information, Table S2).

A drastic change occurred when the leaves reached totally desiccated state (~13% RWC). The absorbance at 1200 nm, 1450 nm and 1930nm increased, while at 1346 nm decreased considerably. The broad band around 1930nm was divided into two peaks centered on 1932nm and 2076 nm. In addition, two new peaks appeared at 1730nm and 2310 nm, related to hydrocarbons^40^. In contrast, variations of the spectra during desiccation of *D*. *eberhardtii* leaves could not be so easily observed nor it was possible to discern any trend in the changes at the water bands dependent on desiccation or rehydration duration. Similarly, during rehydration, it was far easier to observe changes in *H*. *rhodopensis*. The decrement and increment behavior at 1346 nm, 1450 nm, 1790nm and 1930nm water absorbance bands during rehydration process of *H*. *rhodopensis* was totally opposite to that of the desiccation process. The band at 1200 nm showed a clear absorbance decreasing tendency (bottom left inset, Fig. 2). Rehydration process resulted in random fluctuations of absorbance at the seven water absorbance bands in spectra of *D*. *eberhardtii*.

### Spectral Subtraction

**Figure 3:**
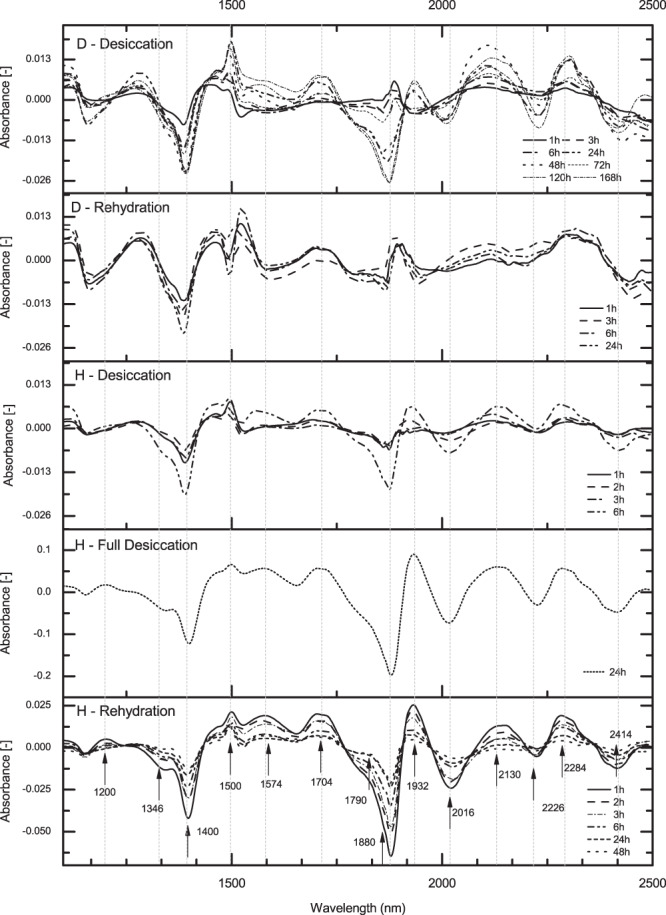
Difference spectra of the stressed and fresh leaves. Difference spectra after EMSC transformation of *Haberlea rhodopensis* and *Deinostigma eberhardtii* during desiccation and subsequent rehydration and the respective spectra of plants in the fresh state.

One approach to emphasize the subtle changes in water dominated NIR spectra is to perform spectral subtraction^24^. Herein, we subtracted the averaged spectrum of each plant’s fresh leaves from the corresponding averaged spectra of leaves at various time points of dehydration and rehydration (stressed leaves) in order to have a closer look at how each plant reacts to stress (Fig. 3). Thus acquired difference spectra revealed absorbance bands at which major spectral variation occurred during desiccation and rehydration stages. For *D*. *eberhardtii* (Fig. 3, two upper panels) variations during desiccation and rehydration stages compared to the fresh state were within the range of 0.039a.u. Similar to *D*. *eberhardtii*, *H*. *rhodopensis* showed absorbance variations within the range of 0.039a.u. until around 25%RWC. However, at full desiccation, the differences were huge, and within the range of 0.3a.u. During rehydration the differences in *H*. *rhodopensis* were within range of 0.085a.u. The magnitude of spectral changes in *H*. *rhodopensis* in the fully dried stage comparing to its fresh state, as well as during rehydration, suggests that this plant undergoes large water structural reorganization in reaction to water stress, since this particular spectral region is dominated by water overtone and combination bands. In comparison, *D*. *eberhardtii*, which can survive dehydration to only about 35%RWC (“point of no return”) displayed no spectral changes of such magnitude.

Also, while no obvious trend in spectral differences was observed for *D*. *eberhardtii*, in *H*. *rhodopensis*, subtracted spectra showed opposite trend of changes during desiccation and rehydration process at the same 14 absorbance bands (indicated with arrows in Fig. 3), majority of which can be assigned to water (detailed assignments presented in Tables S2 and S3, Supplementary Information).

### Principal component analysis

**Figure 4:**
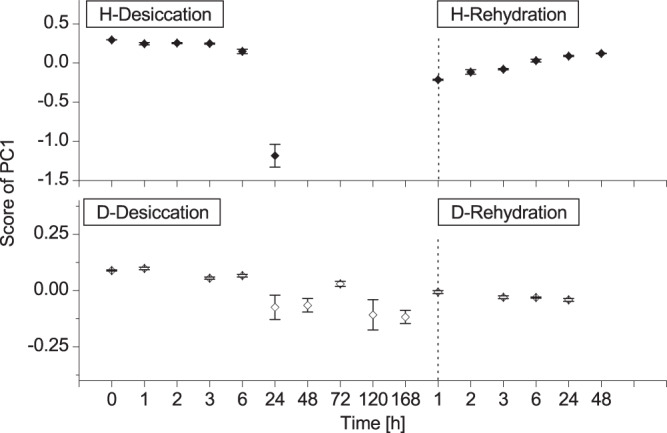
Principal component scores plotted as a function of time. Changes in scores of first principal component (PC1) of *Haberlea rhodopensis* (♦) and *Deinostigma eberhardtii* (◊) during desiccation and subsequent rehydration. Vertical bars indicate standard error of the mean score value for all spectra recorded at specific time point.

**Figure 5:**
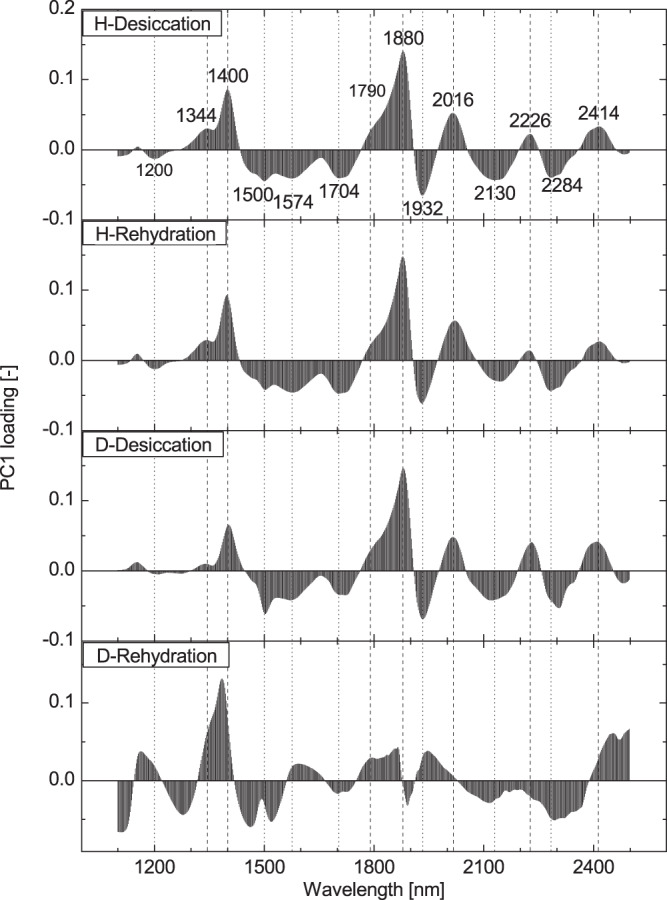
First PC loading for PCA applied to EMSC absorbance data. PC1 loading of *Haberlea rhodopensis* (upper two) and *Deinostigma eberhardtii* (lower two) during desiccation and subsequent rehydration. Dashed and dotted lines indicate the position of positive and negative peaks, respectively, found in *Haberlea rhodopensis* in the desiccation process. Explained variances of each PC1 from top to bottom were 96.1, 88.2, 69.9 and 41.1%, respectively.

Principal component analysis was used to explore in detail spectral variations occurring during desiccation and rehydration process. In *H*. *rhodopensis*, the PC1 scores decreased gradually to about 25% RWC and rapidly declined afterwards (~13% RWC) (Fig. 4). At rehydration, the PC1 scores gradually increased. The PC1 scores during desiccation of *D*. *eberhardtii* resembled the trend of *H*. *rhodopensis*, with small fluctuations, which continued until the plants were fully dried. During rehydration, the scores gradually decreased. PC1 loadings of each treatment and each plant species are plotted in Fig. 5. Variables (wavelength in this case) with high loadings highly contributed to the computation of the PC1 model. There appeared to be 14 characteristic wavelengths over the whole studied NIR range to contribute to the dynamics of the PC1 scores The spectral variation during desiccation and subsequent rehydration process was assigned to O-H vibration in the water (8 out of 14 bands could be assigned to water) and O-H vibration of sugar, N-H vibration of protein, C-H vibration of methylated components, the C = C vibration of alkene, and C-H and C = O vibration of aldehyde (Possible assignments of selected important bands in the PC1 loading are shown in Tables S2 and S3, Supplementary Information).

Explained variances of each PC1 for both plants were substantially different. For *H*. *rhodopensis*, PC1 in both processes explained high and similar percentage of variance: 96.1% and 88.2% for dehydration and rehydration, respectively. For *D*. *eberhardtii*, PC1 explained 69.9% and 41.1%, respectively, which showed nearly 30% less variations explained for dehydration and two times less variations explained by PC1 for rehydration when compared to *H*. *rhodopensis*. Striking was the observation that the PC1 explained variations and loadings for both processes in *H*. *rhodopensis* were almost the same, indicating that dehydration and rehydration processes were reversible in the resurrection plant.

In *D*. *eberhardtii*, the shape of PC1 loading during desiccation showed a similar pattern to that of *H*. *rhodopensis* during desiccation; with some exceptions around 1200, 1400, 1500, 1704 and 2226 nm. However, for *D*. *eberhardtii*, the PC1 loading at rehydration was quite different from that in the desiccation. Selected influential wavelengths for computing the PC1 in desiccation of *D*. *eberhardtii* were the same as for *H*. *rhodopensis*, but those in the rehydration of *D*. *eberhardtii* were entirely different.

### Water molecular dynamics

**Figure 6:**
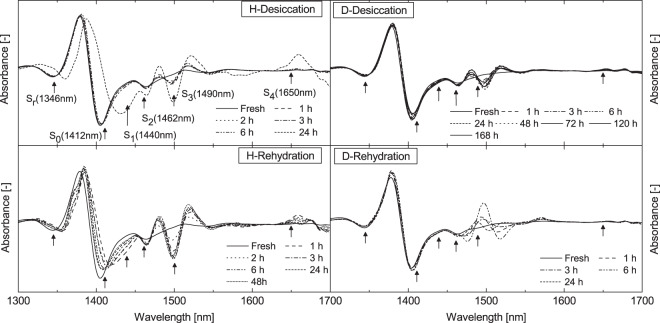
Second derivative absorbance spectra. Second derivative transformed spectra of *Haberlea rhodopensis* (left) and *Deinostigma eberhardtii* (right) during desiccation and subsequent rehydration. Arrows indicate specific wavelengths of water species assigned as S_r_ (1346 nm) – protonated water clusters, S_0_ (1412 nm) – free water molecules, S_1_ (1440 nm) - water molecules with 1 hydrogen bond, S_2_ (1462 nm) - water molecules with 2 hydrogen bonds, S_3_ (1490 nm) - water molecules with 3 hydrogen bonds, and S_4_ (1650nm) - water molecules with 4 hydrogen bonds^42,43^.

Although the NIR spectra look simple, they are very complex consisting of many overlapping peaks. The band centered at 1450 nm, where the water first overtone of stretching vibration mode appears^33,41^ was well studied in respect to water structure or water species. For this reason we calculated the second derivative absorbance spectra in the 1300–1700 nm region (Fig. 6) to resolve the overlapped peaks and facilitate the determination of peak positions.

In the second derivative absorbance spectra of *H*. *rhodopensis* and *D*. *eberhardtii* six peaks were clearly observed where highest spectral variation occurred during dehydration and rehydration processes (marked with arrows in Fig. 6).

These peaks were assigned to the absorption of S_r_ (1346 nm) –protonated water clusters, S_0_ (1412 nm) – free water molecules, S_1_ (1440 nm) - water molecules with 1 hydrogen bond (dimers), S_2_ (1462 nm) - water molecules with 2 hydrogen bonds (trimers), S_3_ (1490 nm) - water molecules with 3 hydrogen bonds, and S_4_ (1650nm) - water molecules with 4 hydrogen bonds^42,43^ (assignments of water species based on the previous studies and the present study are shown in Table S4, Supplementary Information).

**Figure 7:**
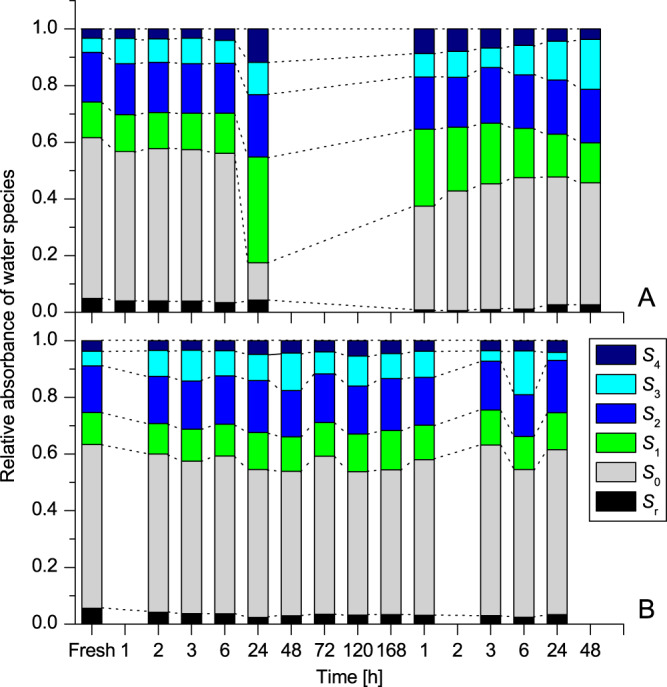
Dynamics of different water species during dehydration and rehydration of *Haberlea rhodopensis* and *Deinostigma eberhardtii*. Relative absorbance of water species in *Haberlea rhodopensis* (**A**) and *Deinostigma eberhardtii* (**B**) during desiccation and subsequent rehydration.

To allow comparison between the water status in plants during dehydration and rehydration, the relative absorbance of 6 water species was calculated as a ration to the sum of all their absorbances at the same time points during dehydration and rehydration (Fig. 7), allowing us to compare their individual contributions (weights) in the water structure respectively.

At control stage (before treatment), the ratio between water molecular species, S_r_: S_0_: S_1_: S_2_: S_3_: S_4_, is quite similar for both plants, suggesting common water structure in the absence of water stress. Proportionally, S_0_ was with the highest, and S_4_ with lowest weight. The first adaptation to desiccation (onset of stress 1 h: ~70%RWC) seemed to be common for both species where S_3_ increases while free water molecules (S_0_) and protonated clusters (S_r_)^44^ decrease. With the progress of dehydration *H*. *rhodopensis* kept all water species in nearly the same ratio until RWC fell to about 20% (6 h). On the contrary, in *D*. *eberhardtii* S_3_ continued to increase and S_0_ to decrease until 45% RWC (3 h). Later on, S_4_ and S_3_ started fluctuating, while S_2_ and S_1_ were constant.

*H*. *rhodopensis* reacted to full desiccation (24 h, 13%RWC) with the increase of S_2_ and S_3,_ but particularly of S_1_ and S_4,_ while S_0_ was drastically decreased. The S_r_ showed gradual decrease during drying. In *D*. *eberhardtii*, both S_4_ and S_3_ have shown small fluctuations with the time.

Taken together, these findings outline major difference in reaction of plants to stress in the terms of water molecular changes. *H*. *rhodopensis* rapidly and readily diminishes RWC, but keeps the water molecular species at the same ratio, while *D*. *eberhardtii* keeps the same high RWC for hours, but ratios of water species fluctuate (see Fig. 1 for comparison).

Approaching the desiccation “point of no return” (48 h, ~35%RWC) *D*. *eberhardtii* showed constant values of S_2_, small fluctuations of S_4_ and S_3_, relative increase of S_1_ and relative decrease of S_0_ and S_r_.

However, there were striking differences between the plants in the relative values of S_0_. While at the end of desiccation in *H*. *rhodopensis* S_0_ was only 13% (down from 57% before treatment) at the “point of no return” for *D*. *eberhardtii* it was 52% (compared to 58% at control stage) of all the water molecular species - half of all water species. The same, very high proportion of free water molecules and subtle fluctuations in ratios of water species were observed even after “point of no return” when *D*. *eberhardtii* plants reached the values of RWC comparable to fully desiccated state of *H*. *rhodopensis* (120 h: ~12.64% RWC) and also at the final time of measurements (168 h: ~9.11%RWC), thus showing that the fully dried states of resurrection and non-resurrection plant are profoundly different.

The rehydration process also showed differences in water status of different plant species. During the first stage of rehydration until the level of 65–70%RWC was reached, the free water, S_0_, increased, at the expense of S_4_ and S_1_ in *H*. *rhodopensis* and S_3_ in *D*. *eberhardtii*. For *H*. *rhodopensis*, in rehydration process, S_1_ and S_4_ decreased in sharp contrast with *D*. *eberhardtii* where they did not change at all. Interestingly, during dehydration - rehydration cycle the water trimmer (S_2_), remained with nearly unchanged relative values in both plants.

Taking together the data from dehydration - rehydration cycle, we can claim that rearrangement of water molecules in various conformations (S_0_, S_1_, S_2_, S_3_, S_4_ and S_r_) was discovered in both plants as a response to abiotic stress. However, sharp decrease in a number of free water molecules S_0_, together with the increase of the number of water molecules with 4 hydrogen bonds S_4_, and especially massive accumulation of water dimers S_1_, was discovered as a unique property of the resurrection plant only.

## Discussion

From the starting point of the analysis by only comparing the dynamics of RWC in the resurrection and non-resurrection species, it is evident that the two plants reacted to desiccation differently. Starting from the 3^rd^ hour of dehydration it seems that underlying defense mechanisms were directed towards different goals: in *D*. *eberhardtii* the goal was to keep the water, while *H*. *rhodopensis* was losing it readily reaching very low levels (13%) within only 24 h.

Initial comparison of near infrared spectra of plant leaves during dehydration and subsequent rehydration showed that major spectral variation occurred at the water overtone and combination bands. More detailed, principal component analysis revealed drastic changes in *H*. *rhodopensis* at absorbance bands which could be related to sugars, proteins, aldehydes, ribosides and methylated compounds, which together are known to be a part of the extensive defense system in resurrection plants^28^. Simultaneously with this badge of different adaptation actions in the leaf tissue antioxidant system^28^, we noticed drastic changes at water absorbance bands suggesting that water is also involved in adaptation to stress conditions, by undergoing changes of its molecular structure. The results of PCA analysis indicated also reversibility of the water dynamics in *H*. *rhodopensis*, which is reflected in the almost the same percentages of explained variance of PC1 for both dehydration and rehydration process, and almost identical shape of PC1 loading vectors. This finding may indicate reversibility of the structural and morphological changes of leaves in *H*. *rhodopensis*, i.e. that dehydration/rehydration processes occurred without irreversible structural damages, which would be in agreement with the reports that *H*. *rhodopensis* exposed to desiccation stress showed no signs of damage^45^.

As the latest reports have outlined, exploring the behavior of water molecular system in biological organisms can uncover different functionalities of water species and improve our understanding of complex biological processes^46,47^. For example, the cold-tolerance of different soybean cultivars was directly related to their ability to keep the water in leaves in the less hydrogen bonded state thus actively avoiding crystalization^26^. Aquaphotomics study on early detection of quality defects in mushrooms correlated the changes in the increase of weakly hydrogen bonded water and the decrease of strongly hydrogen-bonded water with the disruption of cell walls when mushrooms were subjected to mechanical vibration^31^. Another study concerned with the classification of bacterial strains found that compared to non-probiotic and moderate probiotic bacteria, the group of probiotic bacteria creates higher number of small protonated water clusters, free water molecules and water clusters with weak hydrogen bonds in water systems^48^ i.e. their effect on water structure is similar to the effect of the increased temperature. These studies inspired us to look for the peculiarities of water arrangement characteristic for the resurrection plant which might provide novel clues about the desiccation tolerance.

In this work, aquaphotomics analysis revealed 6 specific water species which undergo major changes in the leaves of both plants during dehydration-rehydration process, but these changes seemed very well-orchestrated and goal-oriented in *H*. *rhodopensis*. The first adaptation to desiccation seemed to be common for both plants where S_3_ increased, while free water molecules (S_0_) and protonated water clusters (S_r_) decreased. Also common trait for both plants during entire dehydration - rehydration cycle - the water trimmer (S_2_), the most common water specie in water surrounding proteins^49^ remained with nearly unchanged relative values. This confirms the importance of water – protein interaction for the living process of the plants. However, prominent difference between the species during dehydration was that, while readily and rapidly losing water (RWC), *H*. *rhodopensis* kept the ratio of all these water species constant. The constancy of the ratios implies certain maintenance of the water molecular structure i.e. the existence of dynamic regulation mechanism directed at keeping water in a certain state, which may be favorable from the aspect of desiccation tolerance.

The most striking characteristic of the resurrection plant was the increase of S_4_ and especially S_1_ at the expense of S_0_ during the final desiccation phase. It is worth pointing out, that non-resurrection plant, at 35% RWC, was at point of no return, with still high percentage of free water molecules which continued even when the plants were fully dried. On the other hand, resurrection plant diminished free water molecules very fast during dehydration process. The role of the free water molecules S_0_, in general, is to support biological, physical and chemical reactions^50^. The maintenance of the substantial weight of free water molecules among other water species in the dehydration process of non-resurrection plant, *D*. *eberhardtii*, might be the reason for the damage of its tissues and inability to counteract dehydration. On the contrary, the drastic decrease of free water, in *H*. *rhodopensis* suggests deviation of the usual metabolism and entering the state of anhydrobiosis. The decrease of free water molecules was also observed during drying process of fruits, and is indicative of the shrinkage in the size of the plants^50^. Along with the decrease in free water, *H*. *rhodopensis* increased the number of hydrogen bonded species – notably those with 4 hydrogen bonds, and of water dimers S_1_ in especially large quantities. Organizing water in hydrogen bonded conformations is consistent with the shrinkage in size during drying, the accumulation of sugars^51,52^, subdivision of the vacuoles^52^ and cell wall folding^4,53^ – major changes which result in the decrease of cell volume and logically to the increase of hydrogen bonded water. The accumulation of sucrose and raffinose was already reported in *H*. *rhodopensis*, as a specific adaptation mechanism to survive rapid dehydration by protecting the membranes and possibly even in assisting in scavenging of reactive oxygen species^28,51^.

In resurrection plants, non-reducing sugars play fundamental role in cytoplasmic vitrification as they are able to form glassy structures through hydrogen bonding interactions^54^ - the predominant sugar involved in this mechanism is sucrose^55–57^. This state of cytoplasm leads to enormous decrease of molecular mobility^54^, effectively preventing conformational changes of proteins and fusion of membranes^58^, ensuring the optimal preservation of molecular structures in the dry state^59–61^. The strengthening of the structure of water by increasing the number of hydrogen bonds is a known effect of “structure makers”, and well recognized and utilized property of sucrose^62^. Raffinose on the other hand, is reported to facilitates vitrification of sucrose thereby promoting its protective effect in *H*. *rhodopensis*^51^. The highest levels of both sucrose and raffinose in *H*. *rhodopensis* were observed in the final desiccation stage^28^, which coincides with the highest number of S_1_ and S_4_ water species observed in this work. This finding implies that sucrose and raffinose work on the promotion of hydrogen bonding in water in the final phase of desiccation. Further research in this direction, with different resurrection plant species, aimed at correlating the levels of these sugars with absorbance of water species could provide more clues about their roles and effect on the water structure of leaves.

The new finding here, is that water dimer seems to be of special importance for the resurrection plant in the desiccated state. At this point it is not possible to say whether increased number of dimers is a mere consequence of biochemical alterations in the leaves of *H*. *rhodopensis* resulting in water structure changes where dimers are particularly increased, or that the dimers are accumulated for specific purposes. There are interesting reports about the role of different water complexes as intermediates in chemical reactions in the atmosphere^63,64^. Water dimers, in particular, were found to have enhanced relative humidity-dependent reactivity^65^ and that they react 3.5·10^5^ times faster than the water monomers in the oxidation reactions due to the lower activation barrier^66,67^. Although it would be difficult to explore role of dimers in metabolism of resurrection plants, certainly further aquaphotomics studies similar to the one performed here, with other resurrection plants species could at least reveal whether accumulation of dimers is a universal phenomenon in all desiccation-tolerant plants.

In summary, for the first time, with NIR aquaphotomics analysis, we were able to describe the changes in water molecular structure during desiccation and subsequent rehydration of *H*. *rhodopensis* as a system control along the variation in biochemical components reported earlier^28,29^. In *D*. *eberhardtii*, however, there was no such orchestrated dynamic control and related complementary changes in the water structure. Observed dynamical control of water structure in the leaves of the resurrection plant, *H*. *rhodopensis*, and especially abundance of specific water structure – water dimer S_1_ during the last stage of desiccation lead us to propose that regulation of water molecular structure is a part of the desiccation tolerance mechanism in the resurrection plant and that water dimers may be of specific importance for the “drying without dying” ability of *H*. *rhodopensis*.

## Methods

### Plant material

The present study employed an already established experimental design where *in vitro* grown plantlets of *H*. *rhodopensis* and *D*. *eberhardtii* were subjected to desiccation and subsequent rehydration under controlled conditions^28,29^. We used *in vitro* propagated *H*. *rhodopensis* and *D*. *eberhardtii* (previously named Chirita eberhardtii) as described earlier^68^. The *in vitro* plants were cultured in a plant growth chamber at 22 °C, 50% RH, 16/8 hours of light/dark photoperiod and a photon flux density of 75 µmol m^−2^s^−1^. Plant materials for measurements of relative water content (RWC) and for spectral measurements were prepared separately. For RWC measurements four biological replicates of each plant species were prepared, while for spectral measurements four *D*. *eberhardtii* and three *H*. *rhodopensis* plants were prepared for each time point during dehydration-rehydration cycle.

The experiment was conducted on well-developed and rooted, about two months old plants.

### Dehydration and rehydration procedure

The desiccation stress and recovery system were performed as described earlier^28^. In brief: plantlets were removed from culture vessels and left to dry to the full air-dry stage in a culture room under controlled conditions. Rehydration was carried out on cotton beds by watering the dried plantlets under the same circumstances. The subsequent rehydration was applied to the fully dried *H*. *rhodopensis* samples maintained under controlled conditions for 7 days (168 h). Further drying did not reduce the water content. The rehydration of *D*. *eberhardtii* plantlets was performed after they were dried to about 35% RWC (48 h), since further water loss led to irreversible damage in the non-resurrection plant. This was the lowest RWC level at which *D*. *eberhardtii* plantlets still recover at rehydration. All further references to recovery for *D*. *eberhardtii* deal with plantlets desiccated to about 35% RWC and then rehydrated.

### Relative Water Content (RWC) Measurements

RWC was measured in detached leaves at various time points of drying and rehydration according to^69^:

1 $${\rm{RWC}} \% =({\rm{FW}}-{\rm{DW}}/{\rm{FTW}}-{\rm{DW}})\cdot 100,$$

where FW is fresh weight, DW is dry weight (measured after drying for 48 hours at 80 °C), and FTW is full turgid weight (measured after the leaves were left in distilled water for 24 hours at 22 °C in the dark). Samples for measuring RWC% were separately prepared from four independent plantlets at every point of desiccation/rehydration procedure. Measurements were repeated three times using different leaves and an arithmetic mean of three measurements and standard error were calculated using Origin Pro8.5 software (OriginLab Corp. Northampton, MA).

The RWC of *H*. *rhodopensis* was followed during the entire drying and the resurrection process, while for *D*. *eberhardtii* during drying process and only immediately upon rewatering (0 h) and 24 h after subsequent rehydration.

### Sample preparation for NIR spectroscopy

**Figure 8:**
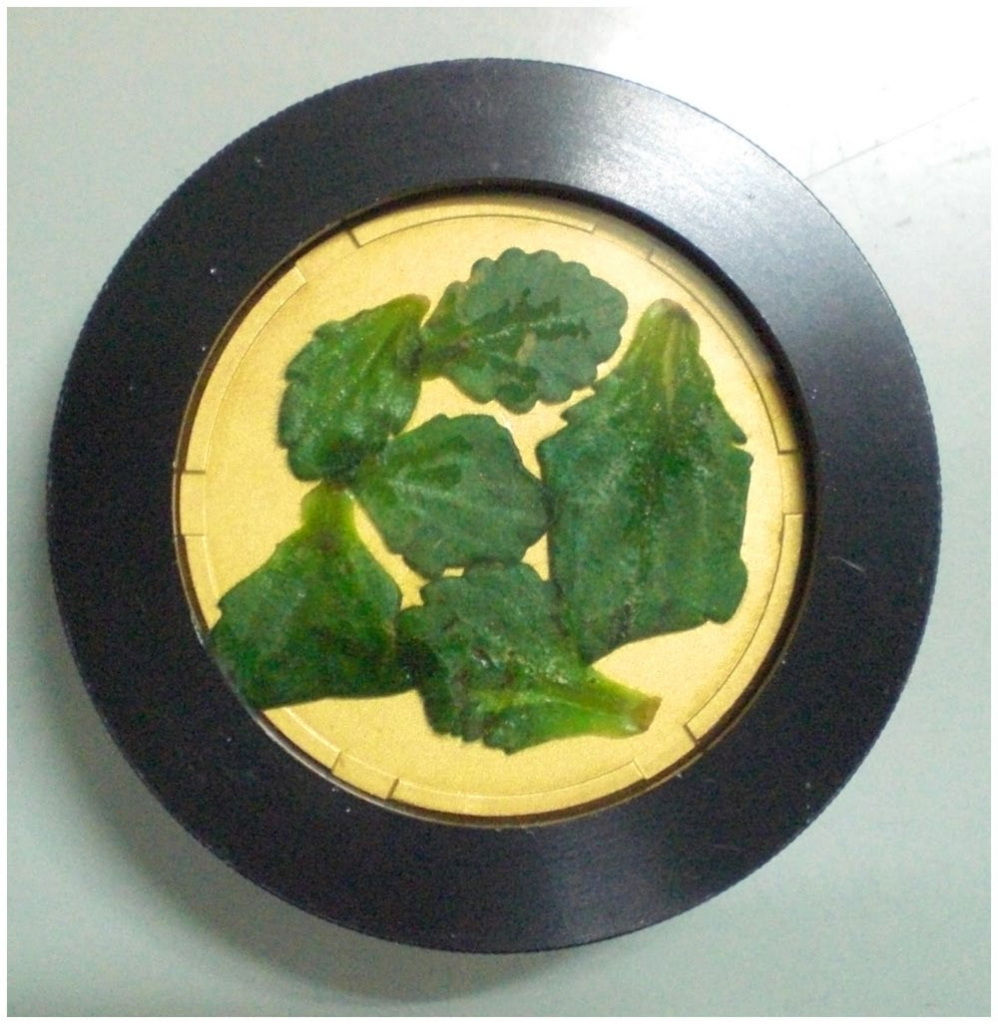
Sample cell for spectral measurement. All leaves of *Haberlea rhodopensis* were set in this case.

Before putting the leaves into the NIR cell, leaves and roots were carefully separated with a surgical knife. A gold reflector was set in the cell; afterward, spectral measurement was carried out (Fig. 8). Transflectance spectra were acquired from 1100 to 2500 nm with 2 nm intervals using NIRSystems 5000 (Foss NIRSystems, MD, USA).

The operating software for spectral data acquisition was WinISI II (version 1.50, Infrasoft International LLC). Each spectrum was a result of the average of 32 scans. For each plant three spectra of different leaves were obtained during measurements. In the desiccation process, the NIR spectra were measured at 0, 1, 2, 3, 6 and 24 hours after starting desiccation for *H*. *rhodopensis* and 0, 1, 3, 6, 24, 48, 72, 120 and 168 hours after beginning desiccation for *D*. *eberhardtii*. In the subsequent rehydration process, the NIR spectra were acquired at 1, 2, 3, 6, 24 and 48 hours after starting rehydration for *H*. *rhodopensis* and 1, 3, 6 and 24 hours after undertaking rehydration for *D*. *eberhardtii*. In total, 274 spectra were acquired for the analysis.

### Spectral data analysis

Multivariate data analysis was carried out using The Unscrambler® X (CAMO, Oslo, Norway). Extended multiplicative scatter correction (EMSC)^70^ was applied for elimination of scattering effects arising from different thickness and surface properties of leaves. All subsequent analysis was performed on EMSC transformed spectral data. Difference spectra were calculated by subtracting the averaged spectrum of each plant’s fresh leaves from the corresponding averaged spectra of leaves at various time points of dehydration and rehydration. Principal component analysis (PCA)^71^ with leave one sample out cross validation was used for exploration of spectral variation during desiccation and subsequent rehydration processes. Norris gap second derivative^72^ (gap size = 3) was further applied to EMSC treated spectra to reveal the presence of hidden and overlapped peaks of the water species.

Further analysis was concentrated on thus found 6 water absorbance bands where particular water species absorb (S_r_, S_0_, S_1_, S_2_, S_3_ and S_4_). In the subsequent analysis in order to investigate dynamics of water species and how much each of the water species contributes to the water molecular structure, the relative absorbance was calculated for each time point during dehydration/rehydration cycle (for the exact times see Table S1, Supplementary Information) as follows:

2 $${A}_{rel,S}=\frac{{A}_{S}}{{\sum }_{i}{A}_{{S}_{i}}},$$

where:

- ${A}_{rel,S}$ - is the relative absorbance of particular water species S (that is, one of the S_r_, S_0_, S_1_, S_2_, S_3_ and S_4_) expressed in percentages,
- ${A}_{S}$ is the mean value of the absorbance for this particular water species found as an arithmetic mean of absorbance for all plant leaves at the wavelength where this water species absorb (for exact wavelengths, see Table S4 and Fig. S1 Supplementary information) and,
- ${\sum }_{i}S$ is the sum of mean absorbances for all the water species S at that time point.

Thus calculated relative absorbances were plotted together on a stacked column chart where each column corresponds to a certain time point during dehydration and rehydration of *H*. *rhodopensis* and *D*. *eberhardtii*. This representation allowed comparison of how the percentages of water species change during water stress and how much proportionally particular water species contributed to the water molecular structure of the plants’ leaves. This data analysis was performed using Origin 8.5.Pro software (OriginLab Corp. Northampton, MA).

## Supplementary information

Supplementary Information

## Data Availability

All datasets generated and/or analyzed during the current study are available from the corresponding authors on request.
