# Author Correction: Water molecular structure underpins extreme desiccation tolerance of the resurrection plant *Haberlea rhodopensis*

**DOI:** 10.1038/s41598-020-66185-5

**Published:** 2020-05-29

**Authors:** Shinichiro Kuroki, Roumiana Tsenkova, Daniela Moyankova, Jelena Muncan, Hiroyuki Morita, Stefka Atanassova, Dimitar Djilianov

**Affiliations:** 1 Laboratory for Information Engineering of Bioproduction, Graduate School of Agricultural Science, Kobe University, 1-1 Rokkodai, Nada, Kobe 657-8501 Japan; 2 Biomeasurement Technology Laboratory, Graduate School of Agricultural Science, Kobe University, 1-1 Rokkodai, Nada, Kobe 657-8501 Japan; 3 Abiotic Stress, AgroBioinstitute, Agricultural Academy, 8 Dragan Tzankov Blvd., 1164 Sofia, Bulgaria; 4 Nanolab, Biomedical Engineering, Faculty of Mechanical Engineering, University of Belgrade, Kraljice Marije 16, Belgrade, 11120 Serbia; 5 Nireco Corporation, 2951-4, Ishikawa Machi, Hachioji, Tokyo Japan; 6 Department of Biochemistry, Microbiology and Physics, Faculty of Agriculture, Trakia University, Stara Zagora, Bulgaria

Correction to: *Scientific Reports* 10.1038/s41598-019-39443-4, published online 28 February 2019

The original version of this Article contained an error in Affiliation 3, which was incorrectly given as ‘Abiotic stress, AgroBioInstitute, 8 Dragan Tzankov Blvd., 1164, Sofia, Bulgaria’. The correct affiliation is listed below:

Abiotic stress, AgroBioInstitute, Agricultural Academy, 8 Dragan Tzankov Blvd., 1164, Sofia, Bulgaria.

This error has now been corrected in the PDF and HTML versions of the Article, and in the accompanying Supplementary Information file.

