# Supplementary material for: Water molecular structure underpins extreme desiccation tolerance of the resurrection plant Haberlea rhodopensis
Source: Sci Rep. 2019 Feb 28;9:3049. doi: 10.1038/s41598-019-39443-4 (PMC6395626; doi:10.1038/s41598-019-39443-4)
Supplement: Supplementary file 1 — Supplementary Information [file 41598_2019_39443_MOESM1_ESM.pdf]

Water molecular structure underpins extreme desiccation tolerance of the resurrection plant *Haberlea rhodopensis*

Shinichiro Kuroki[a,†], Roumiana Tsenkova[b,†,*], Daniela Moyankova[c], Jelena Muncan[d], Hiroyuki Morita[e], Stefka Atanassova[f], Dimitar Djilianov[c,*]

[a]Laboratory for Information Engineering of Bioproduction, Graduate School of Agricultural Science, Kobe University, 1-1 Rokkodai, Nada, Kobe 657-8501, Japan

[b]Biomeasurement Technology Laboratory, Graduate School of Agricultural Science, Kobe University, 1-1 Rokkodai, Nada, Kobe 657-8501, Japan

[c]Abiotic stress, AgroBioInstitute, Agricultural Academy, 8 Dragan Tzankov Blvd., 1164 Sofia, Bulgaria

[d]Nanolab, Biomedical Engineering, Faculty of Mechanical Engineering, University of Belgrade, Kraljice Marije 16, Belgrade 11120, Serbia

[e]NIRECO CORPORATION, 2951-4, Ishikawa machi, Hachioji, Tokyo, Japan, Japan

[f]Department of Biochemistry, Microbiology and Physics, Faculty of Agriculture, Trakia University, Stara Zagora, Bulgaria

[†] Both authors should be considered as first authors

[*] **Corresponding authors:**

Roumiana Tsenkova +81 90-5652-3639, rtsen@kobe-u.ac.jp

Dimitar Djilianov, +359 2 9635413, d_djilianov@abi.bg

Table S1. Dehydration and subsequent rehydration of *Deinostigma eberhardtii* and *Haberlea rhodopensis*. Sample timing and measured RWC content. Empty fields indicate times when measurements were not performed.

	**Dehydration duration [h]**	**RWC [%]**	**Rehydration duration [h]**	**RWC [%]**
Deinostigma eberhardtii	0	91.09	1	--
	1	71.13	2	--
	2	--	3	--
	3	45	6	--
	6	45.18	24	--
	24	43.75	48	83.35
	48	34.78	--	--
	72	22.58	--	--
	120	12.64	--	--
	168	9.11	--	--
	**Dehydration duration [h]**	**RWC[%]**	**Rehydration duration [h]**	**RWC [%]**
Haberlea rhodopensis	0	93.37	1	56.48
	1	72.6	2	56.12
	2	57.19	3	55.63
	3	36.19	6	67.13
	6	24.85	24	90.65
	24	13	48	90.75
	48	12.85	--	--
	72	12.85	--	--
	120	12.85	--	--
	168	12.85	--	--

Table S2. Assignments of the NIR vibrational absorption spectrum of water. ν_r, ν_1, ν_2 and ν_3 indicate rotational, symmetric stretching, bending and antisymmetric stretching mode of the water molecule, respectively. a, b are integers, ≥ 0.

	ν_r	ν_1	ν_2	ν_3	Peak position	References
$a + b = 2$		$a\nu_1$ +	ν_2 +	$b\nu_3$	1200 nm	
$a + b = 2$		$a\nu_1$	+	$b\nu_3$	1450 nm	[1–7]
$a + b = 1$		$a\nu_1$ +	ν_2 +	$b\nu_3$	1930 nm	
$a + b = 1$	ν_r +	$a\nu_1$	+	$b\nu_3$	1346 nm	[8]
$a + b = 1$	ν_r +	$a\nu_1$ +	ν_2 +	$b\nu_3$	1790 nm	

Table S3. Potential band assignments of important wavelengths in difference spectra (Fig.3) and PC1 loading vector (Fig.5).

Wavelength	Potential assignments
1400 nm	Water confined in the local field of ions[9]
1500 nm	1500nm - 1st overtone of the NH stretching mode[3,7,10]
	1500nm - 1st overtone of the OH stretching mode [3,7,10]
	1506nm – protonated water clusters [H+· $(H_2O)_6$] - H_2O in H_5O_2+ asymmetric stretching, 1st overt.[11]
1574nm	Associated O-H str., 1st overtone (sugar)[12,13]
1704nm	C-H stretch, 1st overtone (sugar)[12,13]
	1708nm- H-bonded OH stretching harmonic frequency of H_9O_4+, 1st overtone[14]
1880nm	1876.2nm -OH stretch in fully hydrated hydronium, 1st overtone[15]
	1876.2nm protonated water clusters [H+·$(H_2O)_4$] - H_3O+ asymmetric stretch, 1st overtone [11]
	1880nm - C-H stretch [16]
	1884nm free water molecules (without active H-bonds)[17]
2016 nm	Dissociated O-H stretch + O-H bend. (sugar)[12,13]
	combination of amide A and amide III [6,17]
2130 nm	= C-H str. + C=C stretch (HC=CH)[6,12,13]
	2125nm C-H vibrations in isolated C=C bonds[6]
	2080-2220nm –NH deformation[6]
2226 nm	C-H str. + C=O str. (CHO) [12,13]
2284 nm	C-H str. + C-H bend. (CH_3) [7,12,13]
2414 nm	O-H bend. 2nd overt. (sugar) [12,13]

Table S4. Assignments of the water species in the present study based on previous studies.

Water species	S_r	S_0	S_1	S_2	S_3	S_4	References
Wavelength [nm]	1351	1410	1439	1456	1506	1642	[18]
	1346	1411	1441	1462	1490	1650	[19]
	1346	1412	1440	1462	1490	1650	Present study

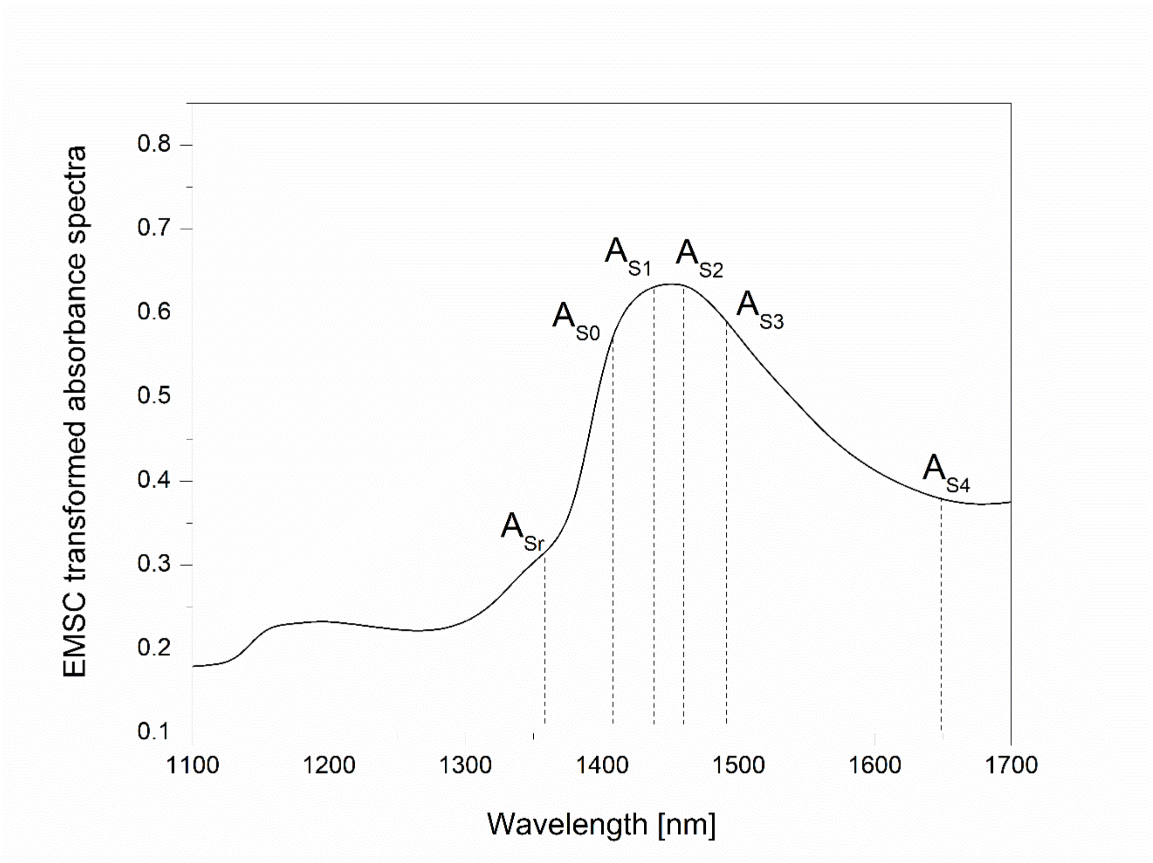

Figure S1. Relative absorbance as a measure of how each water species contributes to the overall water molecular structure is calculated for each time point during dehydration/rehydration cycle as a ration of the mean absorbance of particular water species and the sum of mean absorbances of all 6 analyzed water species (S_r, S_0, S_1, S_2, S_3 and S_4).

Supplementary References

1. Büning-Pfaue, H. Analysis of water in food by near infrared spectroscopy. *Food Chem.* **82,** 107–115 (2003).

2. Luck, W. A. . Structure of Water and Aqueous Solutions. in *Proceedings of the International Symposium Marburg* (ed. Luck, W. A. P.) 248–284 (Verlag Chemie, 1974). doi:10.1002/bbpc.19760800719

3. Workman, J. J. & Weyer, L. *Practical Guide to Interpretive Near-Infrared Spectroscopy. By Jerry Workman, Jr. and Lois Weyer.* (CRC Press, 2008).

4. Iwamoto, M., Uozumi, J. & Nishinari, K. Preliminary investigation of the state of water in foods by near infrared spectroscopy. in *Proceedings of International Near Infrared Spectroscopy / Near Infrared Technology Conference* (eds. Hello, J., Kaffka, K. & Gonczy, J.) 3–12 (Akademiai Kiado, 1987).

5. Kawano, S. & Iyo, C. in *Useful and Advanced Information in the Field of Near Infrared Spectroscopy* (ed. Tsuchikawa, S.) 107–121 (Research Signpost).

6. Nielsen, S. *Food Analysis*. (Springer, 2014).

7. *Near-infrared spectroscopy: principles, instruments, applications*. (John Wiley & Sons, 2008).

8. Ellis, J. W. & Bath, J. Modifications in the Near Infra-Red Absorption Spectra of Protein and of Light and Heavy Water Molecules When Water is Bound to Gelatin. *J. Chem. Phys.* **6,** 723–729 (1938).

9. Kojić, D., Tsenkova, R., Tomobe, K., Yasuoka, K. & Yasui, M. Water Confined in the Local Field of Ions. *ChemPhysChem* **15,** 4077–4086 (2014).

10. Ozaki, Y., McClure, W. & Christy, A. *Near-infrared spectroscopy in food science and technology*. (John Wiley & Sons, 2006).

11. Headrick, J. M. *et al.* Spectral Signatures of Hydrated Proton Vibrations in Water Clusters. *Science (80-.).* **308,** 1765–1769 (2005).

12. Osborne, B., Fearn, T. & Hindle, P. *Practical NIR spectroscopy with applications in food and beverage analysis - UCL Discovery*. (Longman Scientific and Technical, 1993).

13. Williams, P. & Norris, K. H. *Near infrared technology in the agricultural and food industries*. (American Association of Cereal Chemists, 1987).

14. Ojamäe, L., Shavitt, I. & Singer, S. J. Potential energy surfaces and vibrational spectra of H5O2+ and larger hydrated proton complexes. *Int. J. Quantum Chem.* **56,** 657–668 (1995).

15. Relph, R. A. *et al.* How the shape of an H-bonded network controls proton-coupled water activation in HONO formation. *Science* **327,** 308–12 (2010).

16. Chalmers, J. M. & Griffiths, P. R. *Handbook of vibrational spectroscopy*. (J. Wiley, 2002).

17. Alomar, D., Fuchslocher, R. & Stockebrand, S. Effects of oven- or freeze-drying on chemical composition and NIR spectra of pasture silage. *Anim. Feed Sci. Technol.* **80,** 309–319 (1999).

18. Maeda, H., Ozaki, Y., Tanaka, M., Hayashi, N. & Kojima, T. Near Infrared Spectroscopy and Chemometrics Studies of Temperature-Dependent Spectral Variations of Water: Relationship between Spectral Changes and Hydrogen Bonds. *J. Near Infrared Spectrosc.* **3,** 191–201 (1995).

19. Segtnan, V. . *et al.* Studies on the Structure of Water Using Two-Dimensional Near-Infrared Correlation Spectroscopy and Principal Component Analysis. *Anal. Chem.* **73,** 3153–3161 (2001).
